# Acknowledgment to the Reviewers of *Insects* in 2022

**DOI:** 10.3390/insects14020105

**Published:** 2023-01-19

**Authors:** Insects Editorial Office

**Affiliations:** MDPI AG, St. Alban-Anlage 66, 4052 Basel, Switzerland

High-quality academic publishing is built on rigorous peer review. *Insects* was able to uphold its high standards for published papers due to the outstanding efforts of our reviewers. Thanks to the efforts of our reviewers in 2022, the median time to first decision was 16 days and the median time to publication was 39 days. Regardless of whether the articles they examined were ultimately published, the editors would like to express their appreciation and thank the following reviewers for the time and dedication that they have hown *Insects*:
Aaron D. GrossKaren Angeliki KrogfeltAaron DickeyKaren M. VailAaron R. AshbrookKaren MenuzAbdelmutalab AzragKaren RihaniAbdiel Martín-ParkKarin Bakran-LeblAbdou Azaque ZouréKarina WieczorekAbdul Hafiz Ab MajidKarishma ModyAbdulaziz Saad AlqarniKarl ThunesAbdulrahim AlkassabKarla AddessoAbdulrahman S. AldawoodKarol GiejdaszAbid HussainKatarina MladenovicAbigail W. BighamKatarzyna GolanAbraão Almeida SantosKatarzyna KmiećAcebes-Doria AngelitaKate M. BarnesÁdám EgriKatherine NagelAdam James BrunkeKathleen MartinAdam PymKathrin BrownAdam RomanKatja KalanAdam StroińskiKatrin VogtAdam TofilskiKatsuhisa OzakiAditi KulkarniKatsuya IchinoseAdrian ŁukowskiKazuhiro TanakaAdrian SmolisKazuki MiuraAdriana Cristina UrcanKe DongAdrien FónagyKebin LiAgata KaczmarekKeira LucasAgatino RussoKeith F. A. WaltersAgnieszka Kaczmarczyk-ZiembaKelly S. JohnsonAhmed HasaballahKelsey E. FisherAi KitazumiKen-ichi HaranoAijun ZhangKenneth OlsonAjda MoškričKent S. ShelbyAkihiro MiyauchiKentaro ArikawaAkira OtukaKeshava MysoreAkiya JourakuKevin CloonanAlan DorinKevin NjaboAlbane VilarinoKevin R. HinsonAlbert J. AugusteKhalid HaddiAlberto BallerioKim A. HoelmerAlbino BentoKim A. MedleyAlden EstepKim JensenAlec GerryKishan SambarajuAlejandro Del Pozo-ValdiviaKi-Woong NamAleksandra KilianKiyonori KawasakiAleksandra ŁangowskaKiyoshi SaigusaAleksandra TorbicaKlaus H. HoffmannAles BucekKlemen ČandekAleš GregorcKohei OguchiAlessandro GrapputoKoji TsuchidaAlex Bruno Lobato RodriguesKok-Boon NeohAlex DittrichKonrad FiedlerAlexander AlekseevKonstantina ZografouAlexander B. RuchinKostas KougioumoutzisAlexander FaterygaKrijn PaaijmansAlexander G. KirejtshukKris WyckhuysAlexander KirejtshukKrishna KarmakarAlexander KnyshovKristine L. WerlingAlexander KuprinKrisztian MagoriAlexander V. KhramovKrystal R. HansAlexandre De Almeida E SilvaKrzysztof SzpilaAlexandre V. LatchininskyKsenia S. OnufrievaAlexey SolodovnikovKuldeep GuptaAlexis DziedziechKyoichi SawamuraAlfred Edward SzmidtKyung-Hwa ParkAlfredo Jimenez-PerezLadislav ŠimoAlice LacinyLance DurdenAlice MicheluttiLapo RagionieriAlireza SabooriLara MaistrelloAlison GrayLarisa NazarovaAlison R. GerkenLarissa GuillénAlla KolesnikovaLars StraubAllan HruskaLars VilhelmsenAllen CohenLatifa ElhachimiAlmuth HammerbacherLauren WeidnerAlois HonekLaurence A. MoundAlon SilberbushLaurence DinanAlparslan YıldırımLech KarpińskiAlyssa M. WeinsteinLekhnath KafleAmanda CallaghanLenka KouřimskáAmanda D. RoeLeon G. HigleyAmanda PadovanLeon HigleyAmelie HöcherlLeon HugoAmeya D. GondhalekarLeonard B. CoopAmir AyaliLeonard L. DobensAmr A. MohamedLeonardo Bardehle ParraAmr ZaitoonLeonardo L. FrutteroAmy BracciaLeonardo MarianelliAmy FaivreLeopoldo PalmaAmy RodaLevente OrbánAna ButrónLewis Vibul HunAna Guillem-AmatLida FekratAna Luísa Coderniz PicançoLidia LimontaAna PagánLili RenAnastasia CooperLilia AkhmetovaAnastasia NjorogeLilián E. CanavosoAnat Levi-ZadaLina A. GutiérrezAnatoly KrupitskyLincoln SmithAnders AakLinda FieldAndjeljko PetrovićLinda MasonAndre Barreto Bruno WilkeLinde MorawetzAndré Luis Costa-Da-SilvaLing TianAndré Morgado EstevesLiora Shaltiel-HarpazAndrea DragoLisa Jane BirdAndrea JoyceLisa TimmonsAndrea Liliana Clavijo McCormickLiuqi GuAndrea S. SequeiraLiwei WuAndrea SquartiniLiz KoutsosAndrea ToledoLizette L. KoekemoerAndrea TóthováLjubiša StanisavljevićAndreas HoppeLogan W. ColeAndreas KrugerLoganathan PonnusamyAndreas LeclerqueLorella GiovannelliAndreas StarkLorena Barra-BucareiAndrei AlyokhinLorena Ruiz-MontoyaAndrei LegalovLorenzo PrendiniAndrei N. FrolovLorenzo StrattaAndreia BrilhanteLouie YangAndreja Urbanek KrajncLouis Clément GouagnaAndrés Mario VisintínLouis DeharvengAndrew B. NussLuc BelzuncesAndrew CherrillLuc LegalAndrew HaddowLuc SweversAndrew J. RosendaleLuca FinettiAndrew K. JonesLuca RuiuAndrew M. SutherlandLucia ŠmídováAndrew MitchellLuciana TavellaAndrew MoiseffLuciano Veiga CosmeAndrew QuinnLucila ChiffletAndrey FrolovLucy AlfordAndrey GiljovLudvik GomulskiAndrey PrzhiboroLuís BonifácioAndris BukejsLuis De PedroAndrzej BorkowskiLuis EspinoAndrzej GrzywaczLuis F. AristizábalAneta A. PtaszyńskaLuis Gustavo Amorim PessoaAngel GuerreroLuis Mendoza-CuencaAngela BoggeroLuis Miguel De PablosAngela GiangrandeLuís NetoAngela MeccarielloLuis Oswaldo Viteri JumboAngela RoggeroLuisa NardiniAngelos TsikasLuiz Shozo OzakiAnita GiglioŁukasz DepaAnita LiškaŁukasz KajtochAnja MeryandiniLuz Adriana AvilaAnna Cleta CroceMa Carmen BlazquezAnna GajdaMa Cristina Del Rincón-CastroAnna MichalikMaarten J. J. Schrama Anna PapachMacArena GonzálezAnna SeniczakMadhavi KakumanuAnna Voulgari-KokotaMadhumala K. SadanandappaAnnalisa AndreaniMagda Rak CizejAnne FünfhausMaha Mezghani-KhémakhemAnneke A. VeenstraMaisa Da Silva AraujoAntoine FeldenMaja DražićAntoine LecocqMaja Ivana Smodiš ŠkerlAnton E. ShikovMalcolm JoblingAnton SaferMalgorzata Kalandyk-KołodziejczykAntonio BelcariMałgorzata KiełkiewiczAntonio De CristofaroMałgorzata SłocińskaAntonio G. ValdecasasMałgorzata TartanusAntonio J. OrtízMallik MalipatilAntonio ZuritaMamai WadakaAntonios A. AugustinosManoj NayakAntonios E. TsagkarakisManuel Ferreras-RomeroAnyi Mazo-VargasMar Ferrer-SuayArash ZibaeeMarc HerremansAreej Al KhalafMarc KenisArezki IzriMarc S. HalfonArian AvalosMarcel AmichotAriane DorMarcela RodrigueroAriel Ceferino TolozaMarcin KadejAriel Levi SimonsMarcin SkowronekArinder K. AroraMarcio Martinello SanchesArkadiusz UrbańskiMarco InfusinoArmin NamayandehMarco MeneguzArne WeinholdMarco PezziArtai A. SantosMarco PietropaoliArthur G. AppelMarco SalveminiArtur TaszakowskiMarcos Gino FernandesArun RajamohanMarcus Alvarenga SoaresAruna ManrakhanMargarita YavorskayaAsghar TalbalaghiMari H. OgiharaAsif SajjadMaria Aparecida Cassilha ZawadneakAstrid BryonMaria Aparecida FernandezAstrid EbenMaría Buendía-AbadAtashi SharmaMaria C. CarrasquillaAthanasios GiatropoulosMaria Cristina FotiAthanasios KimbarisMaría José BressaAtilano Contreras-RamosMaria José Ladera-CarmonaAtsushi UgajinMaria KonstantopoulouAtul Kumar PandeyMaría L. LópezAtul PandeyMaria Luigia VommaroAugusto LoniMaria MichalczykAulo ManinoMaria Otilia CarvalhoAxel SchwerkMaría Pérez-MarcosAyman MostafaMaria SterzyńskaAyub KhanMaria-Dolores GarciaAzusa KamikouchiMarian J. GiertychBamidele RaheemMariana Guedes CamargoBamisope Steve BamisileMariano Nicolás BelaichBaojian DingMaribel R. PortillaBaojian ZhuMarie C. PizzornoBarbara ContiMarie GuiraudBarbara L. HayfordMarie José DuchateauBarbara LisMarie-Odile Benoit-BiancamanoBarbara ManachiniMarie-Stéphane TixierBarbara Nieradko-IwanickaMarija MiličićBarbara Van AschMarin KovačićBarrett KleinMarina AscunceBeata GabrysMarina J. Orlova-BienkowskajaBeata GrzywaczMarina MazónBeata HoreckaMarina VilenicaBeatrice N. DinghaMarino QuarantaBeatriz WillinkMario Alejandro MarínBen RaymondMario AmatoBenjamin CullMario BjelišBenjamin D. JaffeMário BoieiroBenjamin FuerstenauMario ContariniBenjamin George JacobMaristella MastoreBenoît Sessinou AssogbaMariusz NietupskiBernard StaniecMariusz TrytekBernhard SeifertMarjan KomnenovBert FoquetMark Andrew GillespieBertrand SchatzMark C. HarrisonBethany L. McGregorMark DeryBethany McGregorMark FellowesBharat Bhusan PatnaikMark HarveyBhattarai UpendraMark S. BulmerBilal RasoolMarkéta JanovcováBilali KabulaMarko MutanenBiljana VidovicMarko ProusBin LiangMarkus FranzénBin TangMarkus FriedrichBindiya SachdevMarkus ThammBirgit LockerMarta GariglioBirgit RumpoldMartín AlujaBlanka Premrov BajukMartin FikáčekBo DuMartin H. VilletBoaz YuvalMartin HolmstrupBob MinckleyMartin KuncBong-Kyu ByunMartin ŠigutBouayad NoureddinMartin ŠlachtaBožena BarićMartina BednářováBradley J. SinclairMartina GálikováBrendan HooverMartina Kadoić BalaškoBrenna E. TraverMartina Podnar LešićBrian FedericiMartine A. CollartBrigitte TenhumbergMarwa AttiaBrittany F. PetersonMary Liz JamesonBruninga-Socolar BethanneMarzieh AlizadehBruno BagnoliMasahito T. KimuraBruno MathieuMasako KatsukiBruno RossaroMassimo CristofaroBuddhi AchhamiMassimo MeregalliByju Nambidiyattil GovindanMateusz RawskiCaio Eduardo da Costa DominguesMathilde GendrinCal WelbournMatilde EizaguirreCaleb MartinsMatjaž KuntnerCam DonlyMatteo BracaliniCameron R. CurrieMatteo CalcagnileCameron WebbMatteo MarchioroCamilla R. SharkeyMatthew F. RouhierCarl E. HjelmenMatthias E. SchöllerCarl Scott ClemMattia ManicaCarla Maria PenzMattias ForshageCarlos A. BlancoMaureen Elizabeth WakefieldCarlos BarcelóMauri L. HickinCarlos Felix MarinaMaurizio Francesco BrivioCarlos Holger Wenzel FlechtmannMaurizio MuzziCarlos Lopez-VaamondeMauro Manuel Martinez PachecoCarlos P. SilvaMaxence GerardCarlos Pascacio-VillafánMaysa Tiemi MotokiCarmelo BonsignoreMazhar HussainCarmen CallejasMd Habibullah BaharCarmen PozoMeghan E. DuellCarmen ScieuzoMei Er ChenCarolin HaugMelina CamposCarolina Gomez-DiazMelise C. LechetaCarolina Gracia PoitevinMelissa JordanCaroline KnoxMetka Pislak OcepekCassanelli StefanoMia EeckhoutCatherine LittleMichael A. RiehleCatherine MichauxMichael CaterinoCecilia TamborindeguyMichael DomingueCederic DickoMichael GarveyCees C. Van Den WijngaardMichael H. AllsoppCélia MateusMichael Hilary OtimCengiz KazakMichael I. SitvarinCésar Auguste BadjiMichael J. RaupachCesar Rodriguez-SaonaMichael KošťálChang-hyun KimMichael KristensenChanglong ShuMichael O′DonnellChanglu WangMichael OhlChangqi LiuMichael RedingChao ChenMichael ReiskindChao ShiMichael RoeCharity G. OwingsMichael RustCharl Deacon Charl DeaconMichael San JoseCharles BurksMichael SharkeyCharles J. StuhlMichael SternCharles M MacVeanMichael WinterbournCharles M. OliveiraMichał KrzyżowskiCharles S. AppersonMichal MotykaChee-Seng ChongMIchal ReutCheng ZhangMichal ZurovecChenghao ChenMichelangelo La SpinaChengshu WangMichele RossiniChenyang CaiMichelle FlennikenChenzhu WangMichelle HarveyCherre Sade Bezerra Da SilvaMichelle J. SolenskyCheryl Frank SullivanMichimasa YamasakiChiara FerraciniMidori TudaChiara ScapoliMieczyslawa BogusChieka MinakuchiMiguel A. García-MartínezChihiro HimuroMiguel Ángel Hernández-OñateChloe LahondereMiguel Ángel Navarro-RoldanChong Chin HeoMiguel SimóChris BeattyMihaela KavranChris BosioMilan PernekChris LooneyMilan PlećašChrister HanssonMilena JankowskaChrister WiklundMilyausha KaskinovaChristian J. SchwarzMindi SummersChristian PirkMing BaiChristina PilzMing Der LinChristoph MetzendorfMingkai TanChristoph MusterMingluen JengChristophe Antonio-NkondjioMingshun ChenChristophe BressacMinodora ManuChristopher A. VarnonMirna MihelčićChristopher E. CarltonMiroslav BartákChristopher FosterMiroslawa DabertChristopher GedenMohamed AlburakiChristopher H. DietrichMohamed NasserChristopher M. RangerMohamed Raafat El-GewelyChristopher MayackMohammad Ali Al-DeebChristopher W. WeldonMohammad MehrabadiChristopher WilliamsMohannad IsmailChristos AthanassiouMohsen M. RamadanChristos I. RumbosMokhtar Abdulsattar ArifChuleui JungMolly Duman-ScheelChuntian ZhangMominul AhsanClaire DumenilMonika Riedle-BauerClaudia GarridoMoon Bo ChoiClaudia KeilMoshe Bar-JosephClaudia PașcaMoshe ParnasClaudie DoumsMuhammad FarooqCláudio José Von ZubenMuhammad ShakeelClaus ZebitzMyriam RichaudClement F. KentNabaz R. KhwarahmClive KaiserNabil El-WakeilColince KamdemNancy EndersbyConor FairNaoya OsawaConrad P.D.T. GillettNashwa SallamConsuelo AlmazánNasim ChitsazCorentin JouaultNatalia A. KryukovaCornelis Van AchterbrgNatália RaschmanováCraig A. SchenckNatalia Rosas-RamosCraig MontellNatalia Sawka-GądekCraig R. ChristieNataliia MatushkinaCristian A. VillagraNatasa JokovicCristiano J. De AndradeNathan E. HarmsCristina CarlosNatraj KrishnanCsaba Béla EötvösNaureen RanaDae-Sung HwangboNawaf Al-MaharikDagmara ŻyłaNazar ShapovalDaibin ZhongNazeer AhmedDalibor KodrikNeal T. DittmerDalibor TiteraNeha A. ThakreDalton De Souza AmorimNeil AudsleyDamayanti BuchoriNeil TsutsuiDamien CharabidzeNelson SimõesDan BrabecNesreen M. Abd El-GhanyDaniel Ardisson-AraújoNguyen DuongDaniel DreesmannNicholas C. ManoukisDaniel R. SuiterNicholas JohnsonDaniel RodriguezNicholas M. TeetsDaniel Severus DezmireanNick RuktanonchaiDániel WinklerNicola BodinoDaniela I. AtanasovaNicolas MartinDaniela Magdalena SorgerNicolas MontagneDaniela MarchiniNicolas N. NégreDaniele AlberoniNicolás Pérez HidalgoDaniele CornaraNicole Jean CulbertDaniele Da ReNiels HellwigDaniele De LucaNigel RaineDaniele GiannettiNighat PerveenDaniele Pereira CastroNikita KlugeDaniele SommaggioNikola VesovićDanijela KojićNikolas P. JohnstonDanilo Oliveira CarvalhoNiloufar MahmoudiDanival José De SouzaNils HeinDanon Clemes CardosoNils TjadenDaojun ChengNina A. UshakovaDaria BajerleinNina DevrnjaDarija LemićNoah RoseDariusz J. GwiazdowiczNokkala SeppoDariusz PiesikNooshin SohaniDariusz SkarżyńskiNorbert BeckerDarka HamelNourolah SoltaniDarron CullenNuray BaserDavid A. IngberNurhayat TabancaDavid AlmenarNuria MorfinDavid DeruytterNurit EliashDavid FurthOksana SkaldinaDavid H. HeadrickOle KarsholtDavid HunterOleg AleksandrowiczDavid I. Shapiro-IlanOlga DruzhininaDavid L. HerrinOlga YaroslavtsevaDavid M. WithallOliver KellerDavid MerrittOlivier GnankinéDavid Mota-SanchezOlle AnderbrantDavid OwensOlufemi AjayiDavid Robert HallOsariyekemwen UyiDavid S. HaymerOscar KirsteinDavid TchouassiOwen LonsdaleDavid W. HagstrumOzge Erisoz KasapDavid WariPablo FresiaDavid William InouyePablo Jesús Marín-GarcíaDavide BadanoPablo MontoyaDavide VigettiPairot PramualDean SmithPaloma Martins MendonçaDeby L. CassillPanagiotis IoannidisDedong LiPanagiotis SimitzisDenis J. WrightPanagiotis SkourasDenise BonillaPaola SangiorgioDenise SelivonPaolo FantiDeniz F. ErezyilmazPaolo GabrieliDennis BaulechnerPaolo RaccaDeyu ZouParide DioliDiana MartínPascal DelaunayDiana Perez-StaplesPascal QuernerDiego CejasPasi SihvonenDiego Peres-AlonsoPasquale TrematerraDimitrios AvtzisPatcharin KrutmuangDimitrios KaltsasPatricia CasinoDina CifuentesPatricia DornDina MendonçaPatricia G. TillmanDina Orozco-DavilaPatricia King Jie HungDing WangPatricia LandaverdeDing YangPatrícia Nunes-SilvaDiogo Cavalcanti Cabral De MelloPatrick De ClercqDionyssios Ch PerdikisPatrizia FalabellaDirk AhrensPaul A. AyayeeDiying HuangPaul EadyDmitry KolomenskiyPaul M. AirsDmitry TelnovPaul MasonickDmitry Y. SherbakovPaul-andré CalatayudDomenico BoscoPaulo Fellipe CristaldoDomenico RizzoPaweł AdamskiDominik ChłondPaweł MigdałDonald B. ThomasPedro AbellánDonald C. WeberPedro L. OliveiraDonatella BattagliaPedro SimõesDongjing ZhangPeian TangDonn JohnsonPeng-Cheng LiuDora Aguín-PomboPeng-Fei LuDora HenriquesPerttu SeppaDorcas OrengoPerumal VivekanandhanDoris Lagos-KutzPeter ArmbrusterDoris Rosero-GarciaPeter GreggDorothee TegtmeierPeter H. AdlerDouglas PfeifferPeter KlepsatelDouglass H. MorsePeter MayoEbenezer Antwi GyameraPeter PekinsEbubekir YükselPeter PruisscherEckart StollePeter WitzgallEdgar VilcacundoPeterson Rodrigo DemiteEduard VenterPetr BoguschEduardo FaúndezPetr DolejšEduardo Fuentes-ContrerasPetr PyszkoEduardo Gabriel VirlaPetr ŠípekEduardo Gonçalves Paterson FoxPetr ZemanEduardo MateosPetros DamosEdward WalkerPhilip T. LeftwichEileen KnorrPhilip WardEiriki SunamuraPhilipp E. ChetverikovEkaterina V. GrizanovaPia AddisonEl Desouky AmmarPier Jr MorinElena CargnusPierre OuvrardElena GonellaPing WangElena Iulia IorguPiotr CeryngierElena RomeraPiotr ŚlipińskiEleni I. KoutsogeorgiouPoulami SarkarElgion LoretoPrachurjya DuttaElisabetta GarganiPrashant WaikerElisângela De Souza LoureiroPratik Pravin DoshiElizeu Barbosa CastroPreston ManwillEl-Kazafy Abdou TahaPriyadarshini ChakrabartiEloisa VendemiattiQiao WangElsa Borges SilvaQilian QinElsa Guadalupe Escamilla-ChimalQing YuEman Alaaeldin AbdelfattahQingli ShangEmanuela MauriziQiong YangEmanuele BerrilliQiongbo HuEmiliano MoriQisheng SongEmilie BouhsiraQuanyou YuEmilio HernándezQuentin GuignardEmilio Jorge Tizado MoralesRachael M. HornerEmily A. HartopRachele NieriEmily R. Burdfield-SteelRadomir GraczykEmma L. GillinghamRadomir JaskułaEmmanuel ElangaRafaella Sayuri IoshinoEmmanuel LieanardRafal DlugoszEnric Ureña SalaRafal GosikEnrico De LilloRafał RutaEnrico RuzzierRaffaele GriffoEnrico SchifaniRagnheidur I. ThorarinsdottirEnrique Vargas-OsunaRaisa SukhodolskayaEraldo LimaRajendra AcharyaEran LevinRalf NauenEri Ogiso-TanakaRalitsa BalkanskaEric AgboliRalph Edward DewaltEric ContiRamachandran VinayagamEric JangRamón González-RuizEric RiddickRan WangEric W. ChambersRana Fartab ShoukatErifili P. NikaRandy OliverErika TuckerRangaswamy MuniappanÉrika V. S. AlbuquerqueRanjan PremaratnaErin RehrigRebeca RosengausErin ScullyRegiane Cristina Freitas BuenoErnst WimmerRegiane Maria Tironi De MenezesEsaú Ruiz-SánchezReina TongEsmaeil AmiriRemigiusz GałęckiEsteban Rodriguez-LeyvaRicardo BessinEstefania ContrerasRiccardo FavaroEstève BoutaudRiccardo MorettiEsther LanteroRichard Ian SamuelsEsther U. KadieneRichard KarbanEthel M. VillalobosRichard PaulEustachio TarascoRichard S. ZackEva Sánchez-HernándezRichard W. MankinEvan EschRichou HanEveline C. VerhulstRina HamajimaEverlyne WosulaRinki KumarEvgenyi A. MakarchenkoRinkoo Devi GuptaEwa Pietrykowska-TudrujRita MarulloEwa PuchalskaRo Crystal ChawEwa Szpunar-KrokRobert B. AngusEzequiel GonzalezRobert Baxter SrygleyEzio PeriRobert BodeFabiana SassùRobert D. Mitchell IIIFabio CianferoniRobert FisherFabio Mendonça GomesRobert J. OrpetFabio MosconiRobert JonesFabrizio MontarsiRobert Michael PyleFadia Sara CeccarelliRobert PaxtonFang (Rose) ZhuRobert S. NofemelaFarman AliRobert Warren BehleFatehi FoadRoberta FoligniFatemeh GanjisaffarRoberto Antonio PantaleoniFederica TalaricoRoberto CaldaraFederico CappaRoberto RomaniFederico LessioRobin KundrataFederico PlazziRobin ThomsonFélix PicazoRodrigo ZaluskiFeng CuiRoger A. LaineFerdinand Nanfack MinkeuRoger L. BlackmanFernando De Freitas FernandesRoger NasciFernando MartínRogério SilvaFilip TrnkaRollie J. ClemFilippo FrizziRoman ModlingerFilitsa KaramaounaRomano DallaiFinbarr G. HorganRomeo BelliniFinbarr HorganRomoli OttaviaFiona BrockRomolo FochettiFischer HillaryRonghai HeFlávia J. KrsticevicRosa MurilloFlávio Roberto Mello GarciaRosalba GornatiFlorian KarolyiRosario GilFrancesc Gomez-MarcoRoseli La CorteFrancesc MestresRosemarie C. RosellFrancesca FrentiuRosemarie TedeschiFrancesco CorriasRosilda Mara MussuryFrancesco DefilippoRositsa ShumkovaFrancesco La RussaRoxana Acosta GutiérrezFrancesco NugnesRoy G. Van DriescheFrancesco PorcelliRoy KaspiFrancesco SeverinRoy KirschFrancesco TortoriciRuairi DonnellyFranco MutinelliRuan VeldtmanFrançois DumontRuediger PlarreFrançois LorenzettiRuggero PetacchiFrank ChidawanyikaRui GuoFranklin NyabugaRui-De XueFrantisek RettichRungtip WonglersakFred EllerRunqian MaoFreddie-Jeanne RichardRunzhi ZhangFreddy Ibanez CarrascoRupesh KariyatFreddy TjallingiiRupinder KaurFrédéric FrancisRussell BondurianskyFrédéric LabbéRyan KueselFrederique HilliouSaad ArifFreerk MollemanSaad Naser Al-KahtaniFriederike ReussSabino PachecoFugo TakasuSabrina BertinFulvia BoveraSabrina OlivaFumio YokohariSalih DoğanGábor LöveiSally PaulsonGabor PozsgaiSalmah YaakopGabriel WallauSalvatore BellaGabriele GrecoSalvatore GuarinoGabriele M. BerberichSampat GhoshGabriele RondoniSamuel BrownGabrieli PaoloSamuel H. ChurchGabriella Lo VerdeSamy M. SayedGaëlle J. S. TalrossSamy Mahmoud H. SayedGaku TokudaSandeep JagtapGal RibakSandipa G. GautamGarance Di PasqualeSándor KeszthelyiGareth S. PowellSandra A. AllanGareth ThomasSandra F. BorgesGavin RiceSandra VacasGen KanekoSanford EigenbrodeGenhong WangSantolo FrancatiGenta OkudeSantosh V. RevadiGeoff MorseSanyuan MaGeoff OxfordSaptarshi GhoshGeorg HausnerSara L. BushmannGeorge BroufasSara OppenheimGeorge J. StathasSara RemelliGeorge KennedySarah C. WoodGeorge N. MbataSarah CunzeGeorge PoinarSarah Page LawsonGeorge T. KoliopoulosSarajlić AnkicaGeorge TzotzosSarana SommanoGeorge W. UetzSatoshi HiroyoshiGeorgia KythreotiSatoshi KawakitaGerardo CamiloSaurabh GautamGerardo Sánchez-RojasSayanta BeraGerd GädeScott SalomGero BenckiserSebastian PitaGexia QiaoSenta NiedereggerGéza RipkaSergey BelokobylskijGhislain Tepa-YottoSergey V. KazantsevGil Felipe Gonçalves MirandaSergey Ya ReznikGilbert BarrantesSergey Yu SinevGilberto José De MoraesSergey Yurievich StorozhenkoGimena DellapéSérgio M. OvruskiGino AngeliSergio Sánchez PeñaGiorgia GiordaniSeth J. DormanGiorgio BinelliSeung Ho ChungGiorgio SabellaSeung-Joon AhnGiovanni CiliaSéverin HattGiovanni MessinaShabbir AhmedGiovanni SogariShaku NairGiselher GrabenwegerShaohui WuGissella VasquezSharath Chandra GaddelapatiGiulia LeniSharath GaddelapatiGiulia MagogaSharon AndreasonGiuliana AllegrucciShihuo LiuGiuseppe Eros Massimino CocuzzaShinichi AkimotoGiuseppe Fabrizio TurrisiShinichi TebayashiGiuseppe MazzaShinji NagataGiuseppina PellizzariShipher WuGoran AndrićShirley Vasconcelos KomninakisGordon PortShlomo NavarroGregg HendersonShoaib FreedGrégoire NoëlShruti ShankarGregorio VonoShuhei NomuraGregory A. DahlemShuji ItakuraGregory J. DaglishShuji ShigenobuGregory John RaglandShu-Mei DaiGregory SimmonsShüné V. OliverGregory ZolnerowichShuo WangGrigory PotapovShuo YanGrita SkujienėSigfrid IngrischGritta SchraderSilvia CappellozzaGrzegorz BuczkowskiSilvia Moriano-GutierrezGrzegorz GabryśSilvia Teresa MoraglioGuadalupe RojasSimon BernerGuang XuSimon GalasGuang YangSimon PoppingaGuanhong WangSimona ErricoGuilherme DiasSimona SagonaGunther RaspotnigSimone FattoriniGuobin WangSimone Posedente De LiraGuo-Xing QuanSimone SabatelliGustavo Mora AguileraSin-Ying KoouGustavo Moya-RaygozaSivapong SungpraditGuy SionSnežana RadenkovićGy MakranczySnežana TomanovićGyorgy TurocziSónia DuarteHadassa Yuef Martínez PadrónSonja LeidenbergerHaikou WangSoo Jean ParkHajime YaguchiSoren ToftHakan BozdoğanSoteras FlorenciaHalina KucharczykSoumi MitraHamidou MaiegaSpiridon MantzoukasHamzeh IzadiSpyridon AntonatosHana ŠigutováSrećko B. ĆurčićHanafy IsmailStanislav TrdanHanem KhaterStanislava Chtarbanova-RudloffHanhong XuStanisław CzachorowskiHanna MoniuszkoStefan K. HetzHannah R. Gaines-DayStefania LaudoniaHannalene Du PlessisStefano CivolaniHaobo JiangStefano ColazzaHaralabos TsolakisSteffen WindpassingerHarald LetschStephan KoblmüllerHarald MeimbergStephanie Evelyn KingHarald W. KrennStephen D. GaimariHarpal SinghStephen J. TaerumHarry MeyerStephen JessHassan MajdoubiStephen KluszaHeath OgdenStephen SeatonHeather McAuslaneStephen TrumboHéctor A. VargasSteve WhyardHéctor González HernándezSteven C. CookHeike FeldhaarSteven PresleyHeinz Mueller ShaererStine SlotsboHélcio Reinaldo Gil-SantanaStuart H. McKameyHelena P. FelgueirasSu WangHelene LeBlancSudan GyawalyHélida Ferreira Da CunhaSugey Sinagawa GarcíaHenk BraigSukovata LidiaHenk SiepelSulaiman S. IbrahimHerbert VenthurSumiharu NagaokaHerbert WagnerSunghoon JungHester Elizabeth WilliamsSusan BrownHideharu NumataSusana PascualHidehiro WatanabeSuzanne BlattHidemasa BonoSuzanne KopturHideto HoshinaSwapan ChakrabartyHideyuki ChibaSwapna Priya RajarapuHilary RansonSyed Arif Hussain RizviHiroe YasuiSylvia AntonHiroki SakaiSzymon KonwerskiHiroko SanoTae Kwon KwonHiroyuki YoshitomiTakahiro KikawadaHisashi ÔmuraTakashi MatsumuraHodjat PendarTakeo KuboHong PangTakeru NakazatoHong YangTakeshi MiuraHongbo JiangTakeshi SakuraiHonglin FengTakuma SakamotoHongzhang ZhouTakumasa KondoHossein LotfalizadehTakuya UeharaHouda GharsallahTamar GrossmanHou-Feng LiTamara BotoHoward M. A. ThistlewoodTamara Nunes de Lima-CamaraHuayan ChenTamryn MarsbergHubert CharlesTania IvorraHugh RowellTania YonowHui ChenTatiana A. NovgorodovaHumberto Lánz-MendozaTatsuro KonagayaHüseyin ÖzdikmenTed E. CottrellHyojoong KimTerd DisayathanoowatIan M. ScottTeresa VasconcelosIan MacRaeTerezia HorvathovaIan Robert DadourTeruyuki MatsunagaIbtissem Ben FekihTetsuo GotohIgor PajovicTetsuya KonishiI-Hsin SungThies Henning BüscherIlaria GiovanniniThomas A. O'Shea-WhellerIldefonso Fernández SalasThomas AssmannIlias KioulosThomas C. WebsterIlle GebeshuberThomas DuboisIlya A. Gavrilov-ZiminThomas E. ReaganInaiara De Souza PachecoThomas G. SheltonIndrikis KramsThomas N. VassilakosInga ReichThomas PapeInon ScharfThomas SchmittIrene PicciniThomas SpranghersIrina E. WasserlaufThomas SteinmannIrina G. SinakevitchTiago SimoesIrina HäckerTimm GroutIsaac EsquivelTimothy JuddIsabelle DarbouxTimothy R. FallonIsmail DokerTimothy W. MouralIstván AndóTing LiIvair ValmorbidaTirtza ZahaviIvan DimovTobias LiljaIvan Hadrián TufTodd M. GilliganIvan MilosavljevićTom MatherIvana MajićTom PriceIvana Tlak GajgerTomas CabelloIves HaifigTomáš DitrichIvo E. RigamontiTomáš FialaIvo ToševskiTomas VendlIwona Anna GrussTomoko SteenJ. Joe HullTong BaoJacek FrancikowskiTong ZhangJacek SzwedoTonya BittnerJacinto Benhadi-MarínTonya LanderJack R. BatemanToomas EsperkJacob CorcoranTorranis RuttanaphanJacob WengerTorsten HothornJacqueline HeckenhauerToru TogawaJacqueline V. SarrattToshiharu TanakaJacques DelabieToshko LjubomirovJader de OliveiraTravis Kane JohnsonJaime SanchisTsuneyuki TatsukeJalal Jalali SendiTuan DuongJames B. BurrittUmberto BernardoJames F. WalgenbachUroš GlavinićJames JepsonUrszula WalczakJames M. W. RyallsUwe HombergJames Montoya-LermaVaclav StejskalJames R. HeplerVadim KryukovJames Rudolph MiksanekValentina MastrantonioJames WalgenbachValeria MengaJames ZahniserValeria TaurisanoJan KlečkaVanessa Corby-HarrisJan ŠevčíkVanessa S. DiasJan Van Der BlomVassiliki I. EvangelouJana FančovičováVassilis DourisJane Breen PierceVenera I. TyukmaevaJane CostaVeronica SarateanuJaroslav HolušaVeronika GergócsJarosław BrykVeronika GolyginaJason L. MotternVíctor Rogelio Castrejón-GómezJason M. SchmidtVictoria SorokerJason R. CryanViktor BrygadyrenkoJason SomersViktória TóthJason Sumner-KalkunVillu SoonJean Marcel DeguenonVincent GrayJeane M. C. NascimentoVincent PerrichotJean-François PicimbonVincent PetaJean-Fred FontaineVirginie BoyJedeliza FerraterViridiana Vega-BadilloJeffrey A. ColeVit DvorakJeffrey LittlefieldVivek K. JhaJeffrey OliverVivek KemprajJeffrey R. BloomquistVladimir A. LukhtanovJelica LazarevicVladimír KošťálJendrian RiedelVladimíra KňazovickáJennifer Foltz-SweatVlatka RozmanJennifer KrauelVonnie ShieldsJens AmendtWahid IsraJeongjoon AhnWaldemar KazimierczakJerome A. HogsetteWaqar MajeedJerome Ho Lam HuiWaqas WakilJerome NiogretWee L. YeeJerry ZhuWei DongJerzy A. LisWeiping XuJesper SørensenWeiyi HeJessica D. HohensteinWen LiuJesús A. BallesterosWenchao YangJesús AvillaWen-Jer WuJesus Davila-BarbozaWesley Dondoni ColomboJesus Olivero-VerbelWhitney A. QuallsJevrosima StevanovićWilliam (Bill) KlingemanJhalendra RijalWilliam B. WalkerJian J. DuanWilliam Bart BryantJian YeWilliam D. BrownJianchi ChenWilliam EberhardJiangman HeWilliam GrantJianing WuWilliam MorrisonJiayue YanWilliam Randy MillerJinfeng ChenWilliam TangJing ZhangWilliam WintJing ZhaoWilliam. Wyatt HobackJinglun FengWilson ValbonJinyeong ChoiWojciech Ł. Magowski.Jiraporn RuangsittichaiWojciech NiedbałaJiří KolibáčWolfgang BlenauJiří PatokaWolfram MeyJoan GarrigaWoller DerekJoanna KuliszWoolfolk SandraJoanna WerszkoWopke Van Der WerfJoaquín Guillermo Ramírez-GilWuchun TuJober Fernando SobczakXavier Franch-MarroJocelyn G. MillarXavier PonsJohannes Human GiliomeeXesús FeásJohannes L. M. SteidleXiang LiJohannes StraussXiangbing YangJohn AcornXiang-Jun RaoJohn Allen ShueyXiao ChenJohn C. MorseXiaofei TuJohn C. WiseXiaojiao GuoJohn E. BanksXiao-Long LinJohn H. CroweXiaomao WuJohn J. BeckXiaonan FuJohn J. ObryckiXiaoxia XuJohn K. MoultonXiao-Yong LiuJohn M. LeavengoodXimena PorcasiJohn McCreadieXingeng WangJohn Nzioka MuthamaXingmiao ZhouJohn SnyderXingyue LiuJohn T. HuberXinhua DingJohn TrumbleXinru WangJoice Tom JobXiong-Bing TuJolanta Broz̃ekXiujun WenJonas Hansen KymreXuankun LiJonas WolffXuejun SunJonathan DarbroXueyan LiJonathan GershenzonXun LiaoJonathan WillowYaghoub FathipourJong-Kook JungYahana AparicioJordan M. MarshallYa-Hui ChouJorge CancinoYang LiuJorge Contreras-GarduñoYanli CheJörn BuseYasmin AkhtarJörn TheuerkaufYasuhiko MatsumotoJose A. Jurado-RiveraYasunari MatsuzakaJose Alberto Pinheiro MarcelinoYeon Jae BaeJose Alejandro Martinez-IbarraYetenayet Bekele TolaJosé Bernal del NozalYi-Chang LiaoJosé Bruno MalaquiasYidong YuJose E. PietriYi-jing CenJosé Enrique González-ZamoraYing YanJosé FrancoYinghua HuangJosé Humberto Valenzuela-SotoYingjun CuiJosé Lino-NetoYinping LiJosé López ColladoYoko InuiJosé MarcelinoYong-Bao PanJosé SerrãoYongBiao LiuJoseph S. ElkintonYongjie WangJoseph WagmanYong-Lak ParkJosh ArnoldYongmo WangJoshua KohnYongqi ShaoJostein KjærandsenYooichi KainohJovana BozicYoonseong ParkJowita DrohojowskaYoshihisa AbeJózsef FailYoshihisa HashiguchiJozsef VutsYoshihito SuzukiJuan FerréYoshinori HatakeyamaJuan GuzmanYousef NaserzadehJuan Monribot-VillanuevaYu Chieh David ChenJuanita Rodriguez ArrietaYuan HuangJudith SmithYuanzhen LiuJuil KimYueping HeJulia FerrariYue-Wen ChenJulia GonzálezYuji YasukochiJulia M. MalyshYuki EshitaJulia V. NikolenkoYuliya V. ZakalyukinaJulián F. HillyerYun MaJuliana Amaka UgwuYunchuan DaiJulie UrbanYun-Ru ChenJulieta BrambilaYuri S. TokarevJuliette PoidatzYuriy IlinskiyJun KobayashiYu-Shin NaiJun XuYuyu WangJunaid Ur RehmanYves AlarieJunheon KimZachary DeVriesJunhong WeiZachary ShafferJunjie GuZbyněk KejvalJustin A. JonesŽeljko TomanovićJustin E. HarbisonZeyang ZhouJustyna MaliszewskaZhanghong ShiJuvenil Enrique CaresZhaozhi LuKacem RharrabeZhen ZouKacie AtheyZhenying WangKaijun LuoZhiqiang LiKaio Cesar Chaboli AleviZhixiang ZhangKakeru YokoiZhiyong XiKallare P. ArunkumarZilá Luz Paulino SimõesKanav KhoslaZlatko PuškadijaKanchon DesmahapatraZoltán S. VargaKangkang ChenZoran StanimirovicKangxu WangZsolt BalintKara Tyler-Julian



